# Considerations for Optimizing Dosing of Immunoglobulins Based on Pharmacokinetic Evidence

**DOI:** 10.3390/antib9020024

**Published:** 2020-06-19

**Authors:** Iftekhar Mahmood, Million A. Tegenge, Basil Golding

**Affiliations:** 1 Division of Clinical Evaluation and Pharmacology/Toxicology, Office of Tissue and Advanced Therapies (OTAT), Center for Biologics Evaluation and Research (CBER), Food & Drug Administration (FDA), 10903 New Hampshire Avenue, Silver Spring, MD 20993, USA; 2 Office of Biostatistics & Epidemiology, Center for Biologics Evaluation and Research (CBER), Food & Drug Administration (FDA), 10903 New Hampshire Avenue, Silver Spring, MD 20993, USA; Million.Tegenge@fda.hhs.gov; 3 Division of Plasma Protein Therapeutics, Office of Tissue and Advanced Therapies (OTAT), Center for Biologics Evaluation and Research (CBER), Food & Drug Administration (FDA), 10903 New Hampshire Avenue, Silver Spring, MD 20993-0002, USA; Basil.Golding@fda.hhs.gov

**Keywords:** immunoglobulins, neonates, primary immune deficiency, clearance, half-life, dose

## Abstract

Immunoglobulins (IGs) are widely used for the treatment of immunodeficiency syndromes and several autoimmune diseases. In neonates, IGs have been used for the treatment of alloimmune thrombocytopenia, in neonatal infections and in the rare cases of neonatal Kawasaki disease. This review aims to examine the various dosing regimens of IGs following intravenous (IV) and subcutaneous (SC) administration, pharmacokinetics (PK) of IGs, and the importance of trough values for the prevention of infections in patients with primary immune deficiency (PID). The review also focuses on the mechanism of catabolism of IGs and the impact on the half-life of IGs. Data and reviews were obtained from the literature and the FDA package inserts. The authors suggest that for dosing, the PK of IGs should be evaluated on the baseline-corrected concentrations since this approach provides an accurate estimate of half-life and clearance of IGs. We also suggest employing clearance as a primary PK parameter for dosing determination of IGs. We suggest that IV dosing would be more effective if given more frequently to adjust for the increased clearance at high doses and because the baseline-corrected half-life is much shorter than the baseline-uncorrected half-life. Regarding SC administration, the dose should be adjusted based on the absolute bioavailability (determined against IV dosing) of the product. Finally, we highlight clinical and PK data gaps for optimum and individualized dosing of IGs.

## 1. Introduction

In 1952, Colonel Ogden Bruton [1] noted the absence of gamma globulin in an 8-year-old male with a history of pneumonia and other bacterial sino-pulmonary infections. The patient was unresponsive to four different pneumococcal antigens. He was given 3.2 g immunoglobulin (IG) subcutaneously at monthly intervals. After the administration of IG, he was free from pneumococcal sepsis for more than a year, whereas he had experienced clinical sepsis at least 19 times in the previous four years.

Immunoglobulin products (IGs) are manufactured from pooled human plasma and contain more than 95% unmodified immunoglobulin G (IgG), which has intact Fc-dependent effector functions. Plasma IGs contain polyclonal antibodies, which in humans have five classes of IGs, known as IgG, IgM, IgA, IgE, and IgD [2]. IgG is the most abundant of these IGs [2]. During the manufacturing process of the IGs, IgG is purified and only trace amounts of other subclasses remain. IG products are widely used for the treatment of immunodeficiency syndromes. There are many off-labels uses of IGs for several autoimmune and neurological conditions [3,4]. In neonates, IGs have been used for the treatment of alloimmune thrombocytopenia [5], as an additional treatment in neonatal infections [6,7], and in the rare cases of neonatal Kawasaki disease [7,8]. 

The demand for IGs is increasing due to better recognition of antibody deficiencies and new indications, especially for autoimmune conditions [9]. Over the years, various routes of administration (intravenous (IV), subcutaneous (SC), and intramuscular (IM)) and dosing regimens have been proposed [9]. It is interesting to note that Bruton also used animal-derived hyaluronidase to facilitate IM administration [10]. Although IM administration is now rarely used except as a single dose for short-term prevention or treatment of specific infectious diseases such as hepatitis A and measles [11]. Some hyperimmune globulins are animal antitoxins and can be injected IM as well [11].

When intravenous immunoglobulin (IVIG) preparations became available, the IV route of administration was preferred because of the larger volumes that could be administered and due to the dosing frequency (every 3 or 4 weeks) which was more convenient and improved patient compliance [11]. Subsequently, subcutaneous immune globulin (SCIG) started gaining momentum for routine replacement therapy in primary immunodeficiencies [11].

The objective of this review is to examine the current practices of immunoglobulin dosing in patients with primary immunodeficiency (PID) following IV and SC administration in light of a different perspective in analyzing pharmacokinetic data from patients receiving IGs. 

## 2. Dosing of Immunoglobulins in Patients with PID

The FDA guidance [12] suggests a dose of IVIG ranging from 200 to 600 mg/kg every 3 or 4 weeks. The FDA guidance notes that clinical trials indicate that within the commonly prescribed IVIG dosage range, subjects with higher trough levels may experience greater protection from bacterial infections. The guidance encourages manufacturers of IGs to study the relationships among dose of the product, trough level, and the risk of serious and non-serious bacterial infections.

Investigational Guidelines for clinical trials and core Summary of Product Characteristics (core SPCs) by the European Medicines Agency (EMA) [13] recommend a starting IVIG dose of 400–800 mg/kg given intravenously once, followed by at least 200 mg/kg given every 3–4 weeks. The dosage interval when steady state has been reached varies from 3–4 weeks. The suggested dose is supposed to achieve a trough level of 5–6 g/L. To reduce the rate of infections, it may be necessary to increase the dosage and aim for higher trough levels (see below).

Core SPCs for SC administration recommends that the IVIG dose regimen should achieve a trough level of IgG (measured before the next infusion) of at least 5–6 g/L. This may need to be divided over several days, with a maximal daily dose of 0.1–0.15 g/kg. After steady state IgG levels have been achieved, maintenance doses are administered at repeated intervals (approximately once per week) to reach a cumulative monthly dose of the order of 0.4–0.8 g/kg. Each single dose may need to be injected at different anatomic sites. 

## 3. Pharmacokinetics (PK) of Immunoglobuins

PK is an integral part of the drug development process and is informative for appropriate dose selection. The PK of IGs is often characterized as plasma total IgG level to derive a plasma concentration-time curve, half-life, area under the curve (AUC_0–t_; AUC_0–infinity_), clearance, volume of distribution, maximum concentration (C_max_) and minimum concentration (C_min_) [12]. The PK of IgG has been extensively studied in adults following IVIG and SCIG administration [8,9,11]. The PK of IgG in some cases have been studied for children >2 years of age [14,15,16,17]. IGs are also used in neonates and infants [8,18] but limited PK data exist for neonates and infants. 

IgG clearance mechanisms are complex. IgG may be eliminated via excretion or catabolism. Since the size of IgG is large, filtration of immunoglobulins by the kidneys is minimal [8]. FcγR on the surfaces of cells, such as macrophages, bind IgG and immune complexes, enabling endocytosis, and catabolism of IgG [9]. Although glycosylation of IgG can effect function and binding of Fc to FcγR, this does not affect PK substantially. However, somewhat increased binding of bigalactosylated IgG to FcRn was observed [10].

The PK of some IgG in adults following IV and SC administration of IG products are shown in Table 1, Table 2 and Table 3. The PK of IgG indicate a long half-life (>20 days), low clearance, and a volume of distribution equal to or slightly greater than blood volume indicating that high concentrations of IgG remain in the blood compartment. The pharmacokinetic studies of these IgGs are generally based on uncorrected baseline concentrations. 

**Figure 1:**
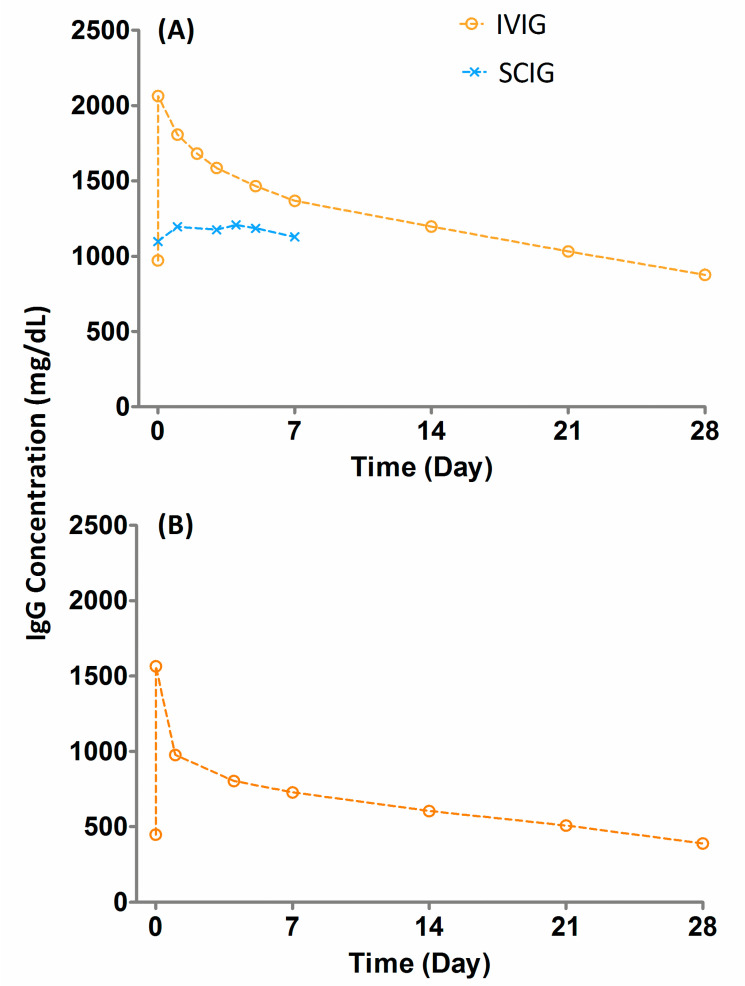
The concentration vs. time profile of immunoglobulin G. (**A**) Concentration vs. time profiles of intravenous immunoglobulin (IVIG) and subcutaneous immune globulin (SCIG) of Gammunex-C in adult subjects and (**B**) IVIG of Gammagard in premature neonates.

The PK studies of these IgGs indicate that the half-lives of IG products are long (>20 days) even though the blood samples are drawn until 21 or 28 days. The reported half-lives of IgG (Table 1) based on uncorrected baseline are generally equal to or greater than the blood sampling time [8,14,15,16,17]. This does not reconcile with the principles of PK. The half-lives of drugs are calculated from the terminal phase of concentration-time data (generally three to four time points) when drug concentrations are gradually declining with a given rate. Like other drugs, the half-life for IgG is also calculated from the terminal phase. For IgG, the terminal phase for the estimation of half-life is generally the baseline values, which barely change over time (Figure 1A,B). Continued sampling below the baseline concentration and without baseline correction leads to an unrealistic long half-life estimation for IgG. In recent years, FDA package inserts for IG products include the PK parameters with and without baseline correction [14,15,16,17]. From these studies, it is obvious that the PK parameters are substantially different between baseline-corrected and uncorrected concentration-time data. The baseline-corrected PK parameters indicate that the half-life of IgG is between 5–7 days, not 21 days (3–4-fold difference), and reconcile very well with the 21 or 28 day blood sampling scheme. Based on pharmacokinetic principles, to accurately characterize the half-life of a drug one should collect blood samples for at least 4 to 5 times the half-life of the drug. In this regard, the PK sampling profiles for IVIG products agree with the half-life values obtained with the baseline correction method. The comparison of half-life values for baseline-uncorrected method following every 3 vs. 4 weeks dosing of IVIG products showed a substantial difference further indicating uncertainty in the PK sampling and half-life values for the baseline-uncorrected method (Table 1). Similarly, the baseline-corrected clearance is 5-fold higher (7.4 vs. 1.5) than the uncorrected clearance (Table 1). 

In children >5 to 12 years of age, the PK of IgG appears to be similar to adults. In children between 2 to 5 years of age, limited PK studies with very small sample sizes (1–3 children per study) exist, hence it is difficult to draw a definitive conclusion on the relationship of the PK of IgG between adults and children in this age group. The PK of IgG is substantially different in preterm and term neonates than adults as shown in Table 2. The half-lives of IgG are similar between the adults and neonates, but clearance is higher (based on per kg body weight) by 1.5–2-fold in neonates compared to adults.

In recent years, the SC administration of IGs has gained momentum. The bioavailability of SCIG is lower than IVIG and hence, the dose of SCIG is generally 1.3 to 1.53 times that of IVIG. IGs following SC administration are generally administered once a week and the dose is adjusted based on the bioavailability and every 3 or 4 week IV dosing. One of the PK characteristics of IgGs following SC administration is that the C_max_ is lower and C_min_ is higher than IV administration (Figure 1 and Table 3). The therapeutic benefit of a high C_max_ folowwing IGIV infusion is not well established. However, C_min_ or trough values play an important role for therapeutic benefit [22,23]. Studies suggest that a minimum C_min_ value of 1000 mg/dL provides protection against infection [23]. 

## 4. Factors Influencing PK and Dosing of Immunoglobulins

Several factors can impact the PK and dosing of IgGs and some of these are mentioned below.

### 4.1. Obesity

The National Center for Health Statistics estimates that for 2015–2016 in the U.S., 39.8% of adults aged 20 and over were obese (including 7.6% with severe obesity) and that another 31.8% were overweight. Obesity rates have increased for all population groups in the United States over the last several decades. The World Health Organization defines obesity as a body mass index of above 30 kg/m^2^ and overweight as >25 kg/m^2^ [24]. The impact of obesity on the PK of IgG is not known. It has been suggested in the literature that obese patients should receive proportionally lower doses of IgG once a certain threshold is reached [24].

A retrospective chart review was conducted at a single center [25]. In this study, there were 173 patients with primary immunodeficiency, including 40 obese patients. The patients received 16% or 20% subcutaneous administration of IgG delivered by infusion pump or subcutaneous rapid push. Based on the findings of the analysis, the author suggested that there was no need for dosing adjustments for SCIG in obese patients. However, the author suggested that the clinicians should use their clinical judgment and experience to optimize dosing of IgG on an individual. The dose should be adjusted to ensure efficacy and safety. 

### 4.2. Precision-Dosing of IgG

In a retrospective cohort study, Stump et al., [26] compared the impact of IVIG dosing using ideal body weight (IBW) or adjusted body weight (ADJBW) with the traditional-dosing strategy (actual body weight (ABW), mg/kg). The patients were diagnosed with hematologic malignancies or underwent hematopoietic stem cell transplant. The primary outcome was infection rate within 30 days of IVIG administration. Secondary outcomes included 60-day infection rate, IgG level response (IgG higher than 400 mg/dL), and the potential of IVIG savings in terms of dose and cost. No differences in infection rate and IgG level response were identified when a precision-dosing strategy was used. Use of a precision-dosing strategy saved $2600/month to the academic medical center where the study was conducted. 

Kuitwaard K et al., [27] conducted a study in 25 patients with chronic inflammatory demyelinating polyneuropathy (CIDP) to determine the variability of serum IgG. The intra-patient variability of the pre-treatment IgG levels, post-treatment levels and increase in serum IgG shortly after IVIG was low (mean CV = 3%, 4%, 10%). The inter-patient variability between patients treated with the same dose and interval was low in pre-treatment, post-treatment IgG level (mean CV = 13%, 11%, 20%). The difference in IgG levels were associated with IVIG dosage (*p* < 0.001). The authors concluded that the low intra- and inter-patient variability in IgG might indicate that constant levels are required to reach this stability.

Table 1 and Table 2 in this manuscript indicate that the variability of PK parameters is not high and IgGs are not highly variable drugs. 

### 4.3. Autoimmunity

In general, IgG doses in autoimmunity are higher (2 g/kg every 4 weeks) than those for PID (400–800 mg every 4 weeks). The mechanism involved in IgG effectiveness in the two conditions are probably different. In PID the treatment is based on antibodies being present that can protect against infectious agents. In autoimmune conditions the mechanism/s are unknown. One possibility is that IgG treatment blocks FcRn resulting in more rapid clearance of all antibodies including those that are causing the autoimmune disease. The role of FcRn in PK of IgG will be discussed more fully below. This would explain why higher doses are required in autoimmune disease compared to treatment of patients with PID. The PK conclusions in this paper from studies in PID patients can probably be extended to auto-immune conditions, namely that more frequent dosing may be beneficial.

### 4.4. Pregnancy

IVIG is generally used in pregnancy for concomitant immunological diseases such as systemic lupus erythematosis, dermatomyositis, antiphospholipid syndrome and fetal alloimmune thrombocytopenia [28,29,30]. 

Unexplained recurrent spontaneous pregnancy loss (RSPL) may occur from an undefined immunological barrier to the normal placenta. Passive immunization with IVIG was found to be promising in uncontrolled trials [31]. The Practice Committee of the American Society for Reproductive Medicine evaluated five randomized controlled trials which assessed IVIG treatment for RSPL [32]. In these five trials, there were 121 IVIG treated patients and 125 placebo-treated patients. The aggregate live birth rate was 62% in the IVIG group and 54% in the placebo-treated controls. This study indicates that IVIG can be beneficial in RSPL.

The impact of pregnancy on the PK of IgGs is not well established; hence, PK studies are needed to optimize antenatal dosing. It is likely that clearance of IgG is increased due to transfer of IgG across the placenta, especially during the latter part of pregnancy.

Ensom and Stephenson [33] conducted a PK study in women with a history of idiopathic secondary recurrent miscarriage or obstetrical antiphospholipid syndrome. The authors’ objective was to make dosing recommendations by comparing IgG concentrations in women receiving IVIG to placebo controls, before and during pregnancy. The enrollment consisted of two groups of women. Women in group A were enrolled for idiopathic secondary recurrent miscarriage (*n* = 25), and women in group B were enrolled in for obstetrical antiphospholipid syndrome (*n* = 10). Of the 35 women in the study, 22 received IVIG 0.5–1.0 g/kg and 13 received the equivalent volume of saline, every 4 weeks from pre-pregnancy until 18–20 weeks of gestation, with dosing adjusted for weight prior to each infusion. There was no significant difference in the pharmacokinetic parameters (C_max_, C_min_, and AUC) within the two subgroups of women receiving IVIG or the control group for the three sampling periods. The authors recommended a weight-adjusted dosage of IVIG during the first and second trimesters to maintain similar drug exposure as seen prior to pregnancy.

Abel et al. [34] reported a statistically significant drop in IgG concentrations throughout pregnancy with the lowest being at term. The authors reported that the IgG concentrations in the first trimester were 18.5 ± 0.88 mg/mL and gradually declined to the second trimester mean concentration of 17.4 ± 0.86 mg/mL. The mean IgG concentration at term was 16.5 ± 0.82 mg/mL.

Schaffer and Newton [35] recommended that the dose of IVIG be increased by 25% during the second trimester. Ledford [36] recommended that the dose of SC or IV gamma globulin be increased approximately 25–50% starting in the late first or second trimester. He suggested to monitor levels in the first and third trimester or more often if there were clinical evidence of infection. He would strive for a level above 800 mg/dL during the third trimester to help ensure an adequate gamma globulin concentration in the newborn. Furthermore, he would reduce the dose immediately after the delivery and recheck a level within 4–8 weeks.

## 5. Discussion

For more than five decades, IGs have been widely used for the treatment of immunodeficiency syndromes and several autoimmune diseases including idiopathic thrombocytopenic purpura in adults and children [3,4,5]. In neonates, IVIG has been used for the treatment of alloimmune thrombocytopenia, infections, and Kawasaki disease. Yet, there is no consensus about the dose, dosing intervals, and the maximum and minimum concentrations of IGs related to therapeutic benefit. It is now widely believed that the trough concentrations of IgG should determine the selection of an IG dose in a given patient [22,23]. This paper focuses on the dosing optimization of IG products for PID patients. 

To establish that certain IgG trough levels are required to minimize infection risk Orange et al. [23] conducted a meta-analysis with all available studies evaluating trough IgG and pneumonia incidence in patients with PID with hypogammaglobulinemia receiving IVIG. Seventeen studies with 676 total patients and 2127 patient-years of follow-up were included. Pneumonia incidence declined by 27% with each 100 mg/dL increment in trough IgG. Pneumonia incidence with maintenance of 500 mg/dL IgG trough levels (0.113 cases per patient-year) was 5-fold higher compared with 1000 mg/dL (0.023 cases per patient-year) trough levels. Based on this meta-analysis, the authors concluded that the risk of pneumonia can be reduced by higher trough IgG levels of at least 1000 mg/dL.

In two clinical studies of a 20% liquid preparation (IgPro20; Hizentra; CSL Behring) conducted in the U.S. and Europe, different doses of SCIG were administered [22]. In the U.S. study (*n* = 38) the mean IG dose was 807 mg/kg/month and was adjusted based on the bioavailability of IVIG observed in the previous IVIG therapy. In the European study (*n* = 46), the mean IG dose was 475 mg/kg/month and was not adjusted for the bioavailability of SCIG from the individual prior monthly doses. Thus, the SCIG dose in the U.S. study was approximately 70% higher than the European study. Patients in the U.S. study achieved higher serum IgG concentrations, spent fewer days per year in hospital (mean = 0.2 vs. 3.48 days) and had fewer missed days of work or school (mean = 2.06 vs. 8 days), and antibiotic treatment was lower in the U.S. study than European study (49 vs. 73 days per patient per year). Overall, this study suggests that higher doses of SCIG are beneficial and the dose of SCIG should be adjusted based on the bioavailability obtained from IVIG administration. In this trial, higher SCIG doses resulted in higher serum IgG concentrations. No corrections were made for individual patient baseline IgG concentration and it is possible that the change from baseline in IgG levels may correlate more accurately with the dose than baseline-uncorrected IgG levels [22].

Another important aspect of IG therapy is the choice of route of administration (IV or SC). An evidence-based review of IGs administered by IV or SC to patients with immunodeficiency was conducted by Lingman-Framme and Fasth [37]. The review was based on 25 studies including: two randomized and 17 non-randomized studies of patients with primary immunodeficiency, one non-randomized study of patients with secondary immunodeficiency, and five studies of health economics. The authors found that IgG trough levels were higher with SC administration than IV administration. The overall conclusions of the authors were that both SC and IV routes of administration of IGs provided protection from serious bacterial infections and were safe. 

IGs are complex molecules consisting of an antigen binding region (Fab domain) and a constant region (Fc domain). IG clearance mechanisms are complex. In the mid-sixties, Brambell [38,39] proposed a mechanism by which IGs were protected from catabolism by binding with receptors located within cellular compartments and/or on the surface of the cells. Brambell receptors were later identified and called neonatal Fc receptors (FcRn) [40]. Studies with knockout mice showed that FcRn receptors are responsible for protecting IGs from catabolism [41,42]. FcRn plays a major role in determining the PK of IgG. After internalization into cells, this Fc receptor protects IgG from degradation in the acidified endosome and facilitates recycling of IgG back into the circulation and explains the relatively long half-life of IgG compared to other plasma proteins. 

Based on the FcRn receptor binding concept, some investigators have tried to show that higher concentrations of IgG are associated with faster elimination rates. The reason for this is that at higher concentrations more free IgG molecules are available to be eliminated due to the saturation of the neonatal receptors. This theory was tested by Schiff and Rudd [43]. 

The authors evaluated the effect of increasing serum concentrations of IgG on the catabolism of IgG. A total of 14 subjects with PID were in the study and they received four doses of IgG as follows:

A mean dose of 100 mg/kg/month (*n* = 14; all subjects received 100 mg/kg/month dose)
A mean dose 157 mg/kg/month (*n* = 14; dose ranging from 100–300 mg/kg/month)
A mean dose 336 mg/kg/month (*n* = 10; dose ranging from 175–690 mg/kg/month)
A mean dose 346 mg/kg/month (*n* = 7; dose ranging from 161–670 mg/kg/month)

Trough concentrations, half-life and clearance of IgG were determined as a function of dose in individual subject. The mean values of these parameters are shown in Table 4. The mean trough values (191 mg/dL to 427 mg/dL) increased with increasing dose. A 3.46-fold increase in dose resulted in a 2.23-fold increase in mean trough concentrations. The mean half-life decreased from 43 days to 33 days (1.3-fold) as a function of increasing dose. The clearance of IgG increased by 1.33-fold from the lowest (100 mg/kg) to the highest (346 mg/kg) dose, indicating that increasing doses of IgG did not have much impact on the clearance. This study does indicate that the elimination of IgG was dose-dependent but in a non-linear fashion. 

**Figure 2:**
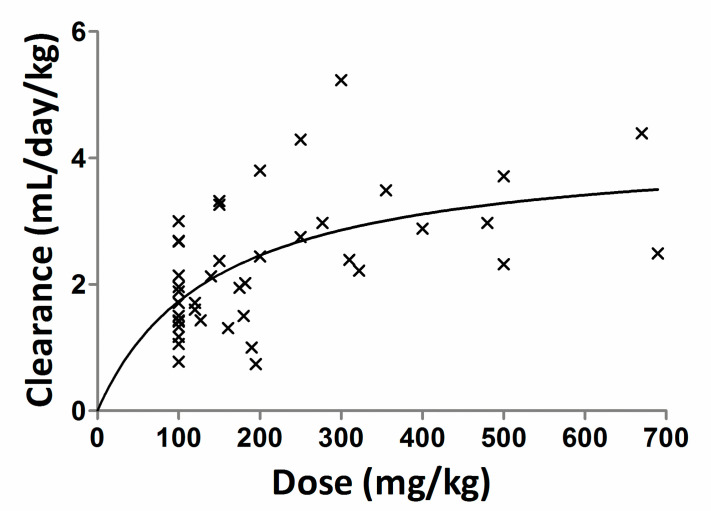
A plot of the relationship between IVIG dose range vs. clearance. The symbol (×) is the observed data and the solid line is nonlinear fit to the data using the Hill equation.

When data were pooled across all doses (*n* = 45 and dose ranging from 100 to 690 mg/kg), clearance increased with dose but the relationship between dose and clearance was weak (*r^2^* = 0.278). In Figure 2, the relationship between dose and clearance is sigmoidal indicating the clearance decreases nonlinearly with increasing dose of IG. Overall, this study does not provide any strong evidence that higher doses of IgG increase the catabolism of IgG. Perhaps the dose range used in this study was not wide enough to evaluate the impact of dose on the catabolism of IgG.

Waldman and Strober [44], established a relationship between the IgG concentration and the fraction of the IgG intravascular pool catabolized per day (Figure 9 in Waldman’s manuscript). In the IgG concentration range seen in patients with PID receiving IGIV, 0–1000 mg/dL, there was a rapid dose-dependent increase in catabolism of IgG. The authors conclude that the most likely explanation of these results is the pathway hypothesized by Brambell based on a protective receptor later elucidated to be FcRn [44].

Thus, higher doses of IGIV are more likely to saturate FcRn receptors. In a study by Fehr et al. [3] in patients with immune thrombocytopenia (ITP) 1–1.5 g/kg doses were associated with increases in platelet count. Hansen and Balthasar showed that IgG infusions in rats with ITP increased platelet counts and reduced the titer of an antiplatelet monoclonal antibody [45]. The authors concluded that this was due to saturation of FcRn receptors and more rapid clearance of the antiplatelet antibody [45]. This conclusion was substantiated by showing that FcRn knockout rats did not show this effect [46]. These findings support the notion that the rapid clearance observed after IVIG administration to PID patients is presumably due to saturation of FcRn receptors.

Based on data submitted to the FDA, with baseline correction of PK parameters, we found that the half-life of IgG is much shorter than previously accepted, namely 5–7 days rather than 21 days. If this is true, dosing need to be adjusted accordingly to achieve optimal trough levels and reduce infection risk. This can be achieved by more frequent dosing, say every 2 weeks instead of every 3–4 weeks.

Future studies leveraging clinical pharmacology tools (e.g., population pharmacokinetics & sparse sampling) can provide quantitative insight on factors affecting the pharmacokinetics of IVIG and SCIG. The knowledge gained from such studies can help to enhance dose individualization of IGs for patients with PID.

## 6. Conclusions

The results of the clinical studies available to date indicate that an optimal dose for IVIG or SCIG is not known [9,11,22]. Factors such as diagnosis and disease conditions of the patients, different dosing schemes and routes of administration, and the criteria for safety and efficacy should be considered for IG dosing. There is also a lack of information relating efficacy or safety with the IgG trough concentrations. A relationship between baseline-corrected IgG levels and dose may be warranted, along with the trough levels [9,22].

Based on baseline-corrected PK data, more frequent IV dosing may be required to achieve optimal trough levels in PID patients. More frequent dosing may also benefit autoimmune conditions where the C_max_ may be more important in lowering the level of unwanted antibodies because of FcRn saturation. 

PK plays an important role in optimizing the dose of a drug in a given patient population. The clearance and trough concentrations of IgG can be used for individual dosing of IVIG or SCIG products. Baseline-corrected concentrations of IgG should be taken into consideration for dosing purposes. It is important to recognize that following monthly administration of IVIG, IgG concentrations are back to baseline concentrations and this may lead to a lack of therapeutic benefit or lack of protection from infections for some patients. Furthermore, considering that binding of IgG to FcRn receptors reduces the catabolism of IgG, higher doses with long dosing intervals may not be an appropriate approach for IgG dosing in PID. The absolute bioavailability (determined against IVIG) is informative for dosing of SCIG. 

Currently, there is a paucity of data in understanding the dose, dosing frequency, effective trough concentration, impact of baseline values, and baseline-corrected IgG concentrations. More work is needed to obtain appropriate pharmacokinetic and clinical data to optimize IVIG and SCIG doses for the patient population with PID. Such studies will help determine whether alternate dosing schedules such as more frequent IV administration at shorter intervals attains higher trough levels and improved clinical outcomes. 

## Figures and Tables

**Table 1:** PK of baseline-uncorrected and baseline-corrected IgG following IV administration.

Products	Half-Life (Days)	Clearance (mL/day/kg)	Half-Life (Days)	Clearance (mL/Day/kg)	Dosing Interval
	Baseline-Uncorrected		Baseline-Corrected		
Asceniv	29 ± 5	1.68 ± 0.40	6 ± 2	9 ± 4	3 weeks
Asceniv	40 ± 12	1.47 ± 0.50	10 ± 8	8 ± 5	4 weeks
Panzyga	32 ± 12	1.44 ± 0.24	5 ± 2	7.2 ± 1.7	3 weeks
Panzyga	45 ± 21	1.44 ± 0.48	8 ± 5	7.4 ± 3.1	4 weeks
Gammaplex 5%	NA	NA	6 ± 6	7.3 ± 6.9	3 weeks
Gammaplex 5%	NA	NA	6 ± 6	5.3 ± 5.5	4 weeks
Privigen	28 ± 6	1.30 ± 0.10	NA	NA	3 weeks
Privigen	45 ± 19	1.30 ± 0.30	NA	NA	4 weeks
Bivigam	20 ± 4	1.97 ± 0.22	NA	NA	3 weeks
Bivigam	33 ± 11	1.41 ± 0.46	NA	NA	4 weeks

NA = data not available; The dose of IV immunoglobulin (IVIG) range from 200 to 800 mg/kg.

**Table 2:** Half-life and clearance of IgG in preterm or term neonates (baseline-uncorrected).

Dose (mg/kg)	Weight (kg)	Age	Half-Life (days)	CL (mL/day/kg)
Sandoglobulin (preterm) [19]				
500	2.6 ± 0.6	35.8 ± 2.9 weeks	11.3 ± 0.6	4.2 ± 1.0
Venoglobulin (preterm) [20]				
1000	1.12 ± 0.21	29.3 ± 1.8 days	19.6 *	5.2 ± 1.5
750	1.06 ± 0.24	28.9 ± 2.0 days	28.7	5.6 ± 0.5
500	1.16 ±0.14	29.2 ± 2.3 days	22.1	3.7 ± 0.8
Gammagard (preterm) [18]				
750	1.09 ± 0.23	28.6 ± 2.9 weeks	22 ± 5	3.4 ± 0.9
500	1.12 ± 0.24	29.2 ± 1.6 weeks	22 ± 6	2.6 ± 0.8
Gamimune [21]				
250 (term)	2.87 ± 0.74	37.4 ± 2.6 weeks	15 **	2.8 **
500 (term)	3.04 ± 0.57	37.6 ± 2.4 weeks	21	4.1
1000 (preterm)	2.52 ± 0.90	34.8 ± 3.0 weeks	35	3.6

* no standard deviation was provided by the authors; ** estimated by the authors of this manuscript from mean concentration-time data hence, no standard deviation. CL: Clearance.

**Table 3:** A comparison of C_max_ and C_min_ of IgG following SC and IV administration.

Products	SC		IV	
	C_max_ (mg/dL)	C_min_ (mg/dL)	C_max_ (mg/dL)	C_min_ (mg/dL)
Cuvitru	1809	1477	2521	1019
Hizentra	1616	1448	2564	1127
CUTAQUIG	1400 ± 440	1200 ± 350	1970 ± 560	1050 ± 260
Gammagard	1393 ± 289	1202 ± 282	2240 ± 536	1050 ± 260
Gamunex-C	NA	1140	NA	958

IV: intravenous; SC: subcutaneous; Cuvitru SC dose was 221 ± 71 mg/kg/week and this was 145% of IV dose given over 4-week period; Hizentra SC dose was 228 mg/kg/week and this was 153% of IV dose given over 4-week period; CUTAQUIG SC dose was 188 mg/kg/week and this was 140% of IV dose given over 4-week period; Gammagard SC dose was 183 mg/kg/week and this was 137% of IV dose given over 4-week periodGamunex-C SC dose was 137% of IV dose of 200–600 mg/kg given over 3 to 4-week period.

**Table 4:** Mean ± SD Pharmacokinetic parameters as a function of immunoglobulin dose.

Dose (mg/kg/Month)	Trough (mg/dL)	Half-Life (Days)	CL (mL/Day/kg)
100	191 ± 112	43 ± 23	1.83 ± 0.64
157	225 ±168	39 ± 14	2.28 ± 1.27
336	390 ± 158	33 ± 9	2.40 ± 0.89
346	427 ± 138	33 ± 16	2.43 ± 1.27

SD: Standard deviation.
